# Supramolecular Co-Assembled Fmoc-FRGDF/Hyaluronic Acid Hydrogel for Quercetin Delivery: Multifunctional Bioactive Platform

**DOI:** 10.3390/foods14152629

**Published:** 2025-07-26

**Authors:** Xian-Ni Su, Yu-Yang Wang, Muhammed Fahad Khan, Li-Na Zhu, Zhong-Liang Chen, Zhuo Wang, Bing-Bing Song, Qiao-Li Zhao, Sai-Yi Zhong, Rui Li

**Affiliations:** 1 Guangdong Provincial Key Laboratory of Aquatic Product Processing and Safety, Guangdong Province Engineering Laboratory for Marine Biological Products, Guangdong Provincial Engineering Technology Research Center of Seafood, Guangdong Provincial Engineering Technology Research Center of Prefabricated Seafood Processing and Quality Control, Guangdong Provincial Science and Technology Innovation Center for Subtropical Fruit and Vegetable Processing, College of Food Science and Technology, Guangdong Ocean University, Zhanjiang 524008, China; m13360352372@163.com (X.-N.S.); 13549158102@163.com (Y.-Y.W.); fahadkhan31202@gmail.com (M.F.K.); zhulina1@stu.gdou.edu.cn (L.-N.Z.); chenzhongliangs123@outlook.com (Z.-L.C.); wz202206@gdou.edu.cn (Z.W.); song@gdou.edu.cn (B.-B.S.); zql2819557995@163.com (Q.-L.Z.); 2 Shenzhen Research Institute, Guangdong Ocean University, Shenzhen 518108, China

**Keywords:** supramolecular co-assembly, delivery carrier, protein-based delivery system, controlled release

## Abstract

**Background:** During food processing and storage, traditional protein-based delivery systems encounter significant challenges in maintaining the structural and functional integrity of bioactive compounds, primarily due to their temporal instability. **Methods:** In this study, a nanocomposite hydrogel was prepared through the co-assembly of a self-assembling peptide, 9-Fluorenylmethoxycarbonyl-phenylalanine-arginine-glycine-aspartic acid-phenylalanine (Fmoc-FRGDF), and hyaluronic acid (HA). The stability of this hydrogel as a quercetin (Que) delivery carrier was systematically investigated. Furthermore, the impact of Que co-assembly on the microstructural evolution and physicochemical properties of the hydrogel was characterized. Concurrently, the encapsulation efficiency (EE%) and controlled release kinetics of Que were quantitatively evaluated. **Results:** The findings indicated that HA significantly reduced the storage modulus (G′) from 256.5 Pa for Fmoc-FRGDF to 21.1 Pa with the addition of 0.1 mg/mL HA. Despite this reduction, HA effectively slowed degradation rates; specifically, residue rates of 5.5% were observed for Fmoc-FRGDF alone compared to 14.1% with 0.5 mg/mL HA present. Notably, Que enhanced G′ within the ternary complex, increasing it from 256.5 Pa in Fmoc-FRGDF to an impressive 7527.0 Pa in the Que/HA/Fmoc-FRGDF hydrogel containing 0.1 mg/mL HA. The interactions among Que, HA, and Fmoc-FRGDF involved hydrogen bonding, electrostatic forces, and hydrophobic interactions; furthermore, the co-assembly process strengthened the β-sheet structure while significantly promoting supramolecular ordering. Interestingly, the release profile of Que adhered to the Korsmeyer–Peppas pharmacokinetic equations. **Conclusions:** Overall, this study examines the impact of polyphenol on the rheological properties, microstructural features, secondary structure conformation, and supramolecular ordering within peptide–polysaccharide–polyphenol ternary complexes, and the Fmoc-FRGDF/HA hydrogel system demonstrates a superior performance as a delivery vehicle for maintaining quercetin’s bioactivity, thereby establishing a multifunctional platform for bioactive agent encapsulation and controlled release.

## 1. Introduction

Some bioactive components in food are unstable and prone to degradation during processing, storage, transportation, and digestion. To enhance stability and bioavailability, some food hydrogels have been developed to encapsulate these unstable compounds [1]. Hydrogel systems, especially those constructed based on biocompatible materials, have demonstrated great potential in protecting active substances. Hydrogels are three-dimensional (3D) polymers that can be engineered to exhibit specific properties and functionalities by carefully selecting design parameters and polymer types (natural or synthetic) and incorporating additional elements such as biomolecules, drugs, and nanomaterials [2]. Hydrogels are capable of absorbing substantial amounts of water, leading to the expansion of their overall structure [2]; therefore, they can serve as excellent candidates for the loading and transport of biomolecules during this expansion process [3]. Moreover, significant advancements in hydrogels have been achieved due to their versatility across a range of potential applications over the past decade, such as in drug administration, tissue engineering, cancer therapy [4], human–machine interfaces [5], and soft robotics [6].

Food proteins possess many functional properties that make them suitable for formulating various nanocarriers, including surface activity, water binding, structuring, emulsification, gelation, and foaming, as well as nutritional aspects [7]. Therefore, protein-based nanocarriers can serve as a platform for delivering bioactive nutrients and have potential applications in extending the shelf life of active substances. Peptides, defined as short-chain protein fragments, can be synthesized at scale while retaining the key structural and functional properties of their parent proteins. Self-assembling peptides (SAPs) protected by 9-fluorenylmethoxycarbonyl (Fmoc) are a novel category of nanohydrogel gelator [8,9]. In recent years, Fmoc-SAPs have attracted great attention in many fields due to their biocompatibility and biodegradability, such as drug delivery and tissue engineering [10,11]. Our previous studies have demonstrated that Fmoc-phenylalanine-arginine-glycine-aspartic acid-phenylalanine (FRGDF) can form nanohydrogels at physiological pH without the need for any crosslinking agents. This occurs through π-β assembly, along with aromatic/electrostatic interactions and hydrogen bonding [12,13]. It is worth noting that this type of supramolecular hydrogel demonstrates unique potential in the field of food functional component delivery: its dynamic, reversible, non-covalent bond network can effectively encapsulate heat/light-sensitive bioactive substances such as vitamins [14]. However, most single-component peptide hydrogels may exhibit transient mechanical properties due to temporal instability. This limitation restricts their effectiveness in drug delivery and dynamic tissue engineering applications.

Natural polysaccharides have garnered significant interest because of their unique characteristics, making them suitable for the improvement of SAP hydrogel properties [15]. Polysaccharides such as fucoidan [16,17], alginate [18], chitosan [19], and glucomannan [20] have been used to form multicomponent networks with SAPs. Hyaluronic acid (HA) is a naturally occurring glycosaminoglycan, characterized as an acidic mucopolysaccharide composed of repeating units of D-glucuronic acid and N-acetylglucosamine [21]. HA has gained recognition as a promising biopolymer for various biomedical applications due to its biocompatibility, biodegradability, and intrinsic ability to interact with cell surface receptors. These attributes render it an appealing candidate for drug delivery systems and tissue engineering applications [22]. For example, HA-based nanodelivery systems such as nanoparticles, nanomicelles, and nanogels have proven effective as appealing carriers for the targeted delivery of bioactive compounds such as polyphenols [23]. However, HA has not yet been utilized for the modification of SAP hydrogels.

Quercetin (Que), a flavonoid classified within the polyphenol group, is identified chemically as 3,3′,4′,5,7-pentahydroxyflavone. It is a naturally available flavonoid, often used as a dietary ingredient and supplement [24]. Que is abundantly present in various natural sources [25] and has been extensively investigated for its properties, which combat inflammation, provide antioxidant effects, fight viruses, and prevent cancer in both animals and humans [26]. These multi-target effects of synergistic interactions give it great appeal in the fields of dietary health care, disease prevention, and adjuvant therapy, and it is regarded as a highly valuable natural active molecule [27]. However, quercetin has poor water solubility [28] and chemical instability [29], resulting in a significant decrease in its bioavailability during food processing, storage, and human digestion [30]. Incorporating Que into suitable therapeutic nano-systems can not only significantly enhance its solubility and physicochemical stability, but also achieve long-lasting and targeted delivery by precisely regulating release kinetics [31].

The interactions between polyphenols, proteins, and polysaccharides involve both covalent and non-covalent bonds [32,33]. These two interaction types significantly affect protein glycosylation, as well as the formation, elasticity, and clarity of gels [34]. Furthermore, the addition of phenolic acids to protein–polysaccharide complexes can enhance their physical and thermal stability, as well as their performance in delivery applications [35]. The properties of the ternary complexes are influenced by the binding sequences of proteins, polyphenols, and polysaccharides [36]. However, the impact of HA on the mechanical properties of the complex and the interactions among Que, peptide, and HA have not been studied to date.

**Figure 1:**
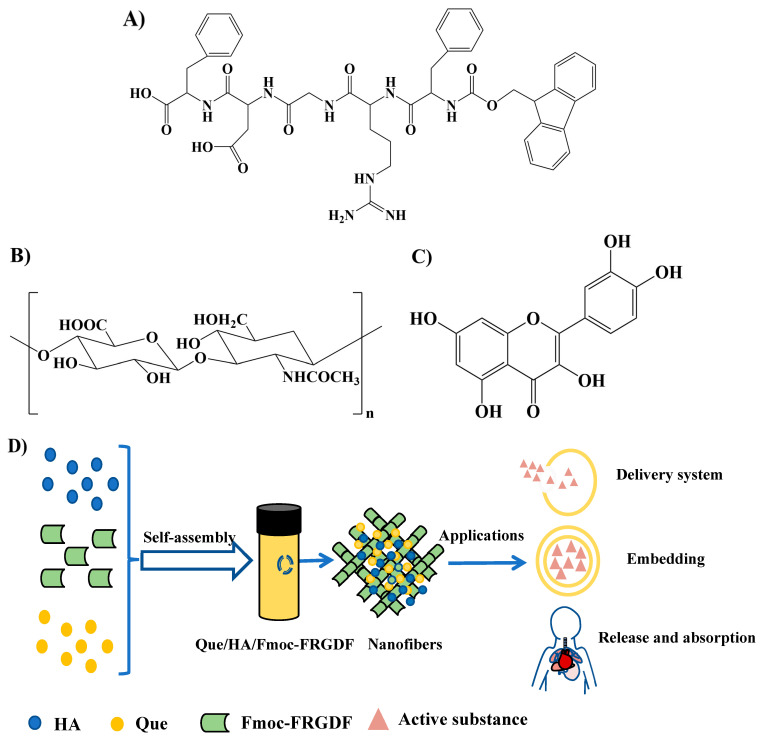
The molecular structures of Fmoc-FRGDF and hyaluronic acid (HA), with a depiction related to the processes of self-assembly and co-assembly that lead to the formation of hydrogels. The chemical structures are depicted for Fmoc-FRGDF (**A**), HA (**B**), and quercetin (Que) (**C**); the self-assembly process of composite hydrogels and the potential application of nano-hydrogels as delivery systems are also shown (**D**).

Therefore, this research employed HA to modify the rheological properties and stability of a self-assembling peptide Fmoc-FRGDF hydrogel, and the resulting composite hydrogel served as a carrier for Que by the formation of a ternary complex nanogel system (Figure 1). The mechanical properties, in vitro degradation behavior, secondary structure, and micro-morphologies were characterized using techniques such as circular dichroism (CD), Fourier transform infrared spectroscopy (FTIR), and transmission electron microscopy (TEM). Additionally, both the encapsulation efficiency and release profile of Que were evaluated. Notably, this study investigated the effects of Que on the rheological properties and microstructure of the ternary complex, as well as the interactions among Que, Fmoc-FRGDF, and HA. This study aims to elucidate the role of HA in modulating the rheological behavior and colloidal stability of Fmoc-FRGDF/HA hydrogels, characterize the polyphenol-induced physicochemical alterations in ternary co-assembled complexes, and probe the molecular-level interactions within the Que/Fmoc-FRGDF/HA system. This discovery elucidates the co-assembly mechanisms governing polyphenol–peptide–polysaccharide interactions, while demonstrating the potential of supramolecular hydrogels as stimuli-responsive platforms for advanced delivery systems. Furthermore, it establishes a theoretical foundation for engineering future protein-based hydrogels with programmable self-assembly and targeted release functionalities.

## 2. Materials and Methods

### 2.1. Material

Hyaluronic acid (HA), obtained from Shanghai McLean Group (Shanghai, China), had a purity of 97.0% and a molecular weight ranging from 40 to 100 kDa. Fmoc-FRGDF was sourced from Pepmic Co., Ltd. (Suzhou, China) with a purity greater than 95.0% and a molecular weight of 863.0 Da. Quercetin (Que) was purchased from Shanghai Aladdin Technology Co., Ltd. (Shanghai, China), exhibiting a purity of 95.0% and a molecular weight of 302.2 Da. Additionally, all other chemicals used, including 0.5 M NaOH, 0.1 M HCl, and 0.1 M potassium phosphate saline (PBS at pH 7.4), were of analytical quality.

### 2.2. HA/Fmoc-FRGDF Gel Preparation

HA in varying amounts (0, 0.1, 0.5, 1.0, and 1.5 mg) was weighed alongside 10.0 mg of Fmoc-FRGDF into separate glass bottles, corresponding to the labels A0 (HA, 0 mg/mL), A1 (HA, 0.1 mg/mL), A2 (HA, 0.5 mg/mL), A3 (HA, 1.0 mg/mL), and A4 (HA, 1.5 mg/mL). To each bottle, we added 400.0 μL of ultrapure water and then introduced 65.0 μL of a 0.5 M NaOH solution while vortexing until fully dissolved. Subsequently, we carefully added drops of a 0.1 M HCl solution while maintaining continuous vortexing. At the end, we incorporated an additional volume of 480.0 μL of a PBS solution to achieve a total volume of exactly 1.0 mL before applying ultrasonic processing to eliminate air bubbles. The samples were allowed to sit undisturbed for approximately 24 h at 25.0 °C for the formation of HA/Fmoc-FRGDF hydrogels.

### 2.3. Que/HA/Fmoc-FRGDF Gel Preparation

For preparing Que-loaded hydrogels, Que (30.8 mg) was first dissolved in NaOH solution to create a Que/NaOH mixture with a concentration of Que set at 2.0 mg/mL. The mixture followed the same procedure used to make the HA/Fmoc-FRGDF hydrogels, in which the final quercetin content was 2.0 mg/mL. The samples were designated as B0 (HA, 0 mg/mL), B1 (HA, 0.1 mg/mL), B2 (HA, 0.5 mg/mL), B3 (HA, 1.0 mg/mL), and B4 (HA, 1.5 mg/mL).

### 2.4. Rheological Properties

The rheological characteristics of the hydrogel samples were evaluated using a rheometer (Thermo Scientific HAAKE MARS III, Karlsruhe, Germany). The experimental procedure was adapted based on our prior research [37]. A sample volume of 1.0 mL was subjected to dynamic oscillation measurements with a 35.0 mm parallel plate (P35 TiSE), maintaining a gap of 0.2 mm. The frequency range applied was from 0.1 to 10.0 Hz, with a strain set at 1.0%, and the tests were conducted at a temperature of 25.0 °C throughout the linear viscoelastic range (LVR) for all samples.

### 2.5. In Vitro Degradation Property

The mechanical stability of the hydrogel structure and the maintenance of physiological pH were assessed, and an in vitro degradation curve was generated. The experimental approach was slightly modified from previous work by Kocak et al. [38]. In this procedure, we combined 2.0 mL of PBS with the samples (A0, A1, A2, A3, and A4) in sealed containers. These containers were then placed in a shaking incubator set at 37.0 °C with a rotation speed of 100.0 r/min. Supernatant samples were gathered at time points of 0, 24, 48, 72, 96, 120, 144, 168, and 192 h, respectively. The remaining hydrogels were gently wiped dry using paper towels and weighed afterward. An equal volume of PBS was added back to each gel sample after weighing to monitor weight loss throughout the experiment duration. The remaining weight ratio of hydrogel (%) was calculated according to Equation (1).

The remaining weight ratio of hydrogel (%) = Wt./W0 × 100%(1)

Wt. represents the mass of the hydrogel that remained following degradation at a specified time interval, while W0 denotes the original weight of the hydrogel.

### 2.6. Fourier Transform Infrared Spectroscopy (FTIR)

An FTIR spectrometer (BRUKER TENSOR II, Ettlingen, Germany) was employed to analyze the functional groups and secondary structures of the samples numbered A0, A1, A2, A3, A4, B2, and B3 within the wavelength spectrum from 4000 to 400 cm^−1^ with a resolution of 4 cm^−1^ over 32 scans. Trace freeze-dried samples were combined with dried potassium bromide in a ground mixture that was then pressed into flake, and pure potassium bromide served as the blank control (with a weight ratio of potassium bromide to sample set at 100:1). The analysis of the hydrogel’s secondary structure focused on the spectral range between 1750 and 1550 cm^−1^ [39].

### 2.7. Circular Dichroism (CD) Spectroscopy

The CD spectra were recorded, employing a Chirascan V100 spectrometer (Applied Photophysics Ltd., Leatherhead, UK). The experimental procedure was adapted from the study conducted by Tai et al. [40]. The samples were diluted to achieve a total polymer concentration of 0.01% by weight. Measurements were carried out at 25.0 °C over a wavelength range from 180 to 320 nm, with a bandwidth set at 1.0 nm.

### 2.8. Transmission Electron Microscope (TEM)

The structure at the microscopic level of the sample was examined employing a JEOL-2100 LaB6 transmission electron microscope (TEM) manufactured by JEOL Ltd. in Tokyo, Japan, following our previously established method [41]. Copper grids with a 200-mesh carbon coating served as the sample holders. The hydrogel was combined with deionized water at a specific ratio of 1:15, subjected to sonication for 5 min, and then filtered twice through a filter membrane. The resulting filtrate was placed onto the copper grid, followed by the application of a drop of 2.0% (*w/v*) phosphotungstic acid for staining, and was allowed to dry at 25.0 °C. The samples prepared were observed at an operating voltage of 120.0 kV. The fiber thickness of the TEM images was quantitatively analyzed by using Fiji ImageJ software.

### 2.9. Assessment of Encapsulation Efficiency (EE%) and Loading Capacity (LC%)

The encapsulation efficiency (EE%) and loading capacity (LC%) of quercetin (Que) were determined by assessing the concentration of free Que in the supernatant. Briefly, the supernatant was obtained through centrifugation at 10,000 g for 10 min. The absorbance of the clear liquid above the sediment was measured at 374.0 nm to quantify the amount of free Que within a range from 2.5 to 100.0 μg/mL (Y = 1.2914x − 0.0032; R^2^ = 1.0000). The calculations for EE% and LC% were performed using Equations (2) and (3).

EE (%) = Que weight in gel (mg)/Total weight of Que (mg) × 100%(2)

LC (%) = Que weight in gel (mg)/the weight of the complex (mg) × 100%(3)

### 2.10. In Vitro Release

Dialysis bags with a molecular weight cutoff of 100–500.0 Da were utilized to evaluate the in vitro release of Que from the composite hydrogel samples. The concentration of Que loaded into the hydrogels was set at 2.0 mg/mL, and the samples were designated as B0 (HA, 0 mg/mL), B1 (HA, 0.1 mg/mL), B2 (HA, 0.5 mg/mL), B3 (HA, 1.0 mg/mL), and B4 (HA, 1.5 mg/mL). Each dialysis bag containing the samples was submerged in beakers filled with 50.0 mL of PBS solution, which were sealed accordingly. These beakers were maintained on a shaking table set at 37.0 °C with a constant rotation speed of 100.0 r/min. At the specified time points of 0, 6, 12, 18, 24, 30, 36, 42, and 48, 2 mL of equally divided PBS solution was extracted and the absorbance was measured at 286.0 nm (Y = 0.1501x; R^2^ = 0.9922). To ensure a consistent volume throughout the experiment, an equal amount of PBS solution was added to each beaker after sampling.

### 2.11. Study on the Release Kinetics of Que

This approach was adapted from the method described by Bialik-Wąs et al. [42] with some minor adjustments. In particular, the release profile of Que from the nanohydrogels could be modeled using different kinetic equations. These included the zero-order kinetic model (Equation (4)), the first-order kinetic model (Equation (5)), and the Korsmeyer–Peppas kinetic model (Equation (6)), which represents the initial 60 wt.% of release behavior for a theoretical distribution.

(4) $$Zero-order\;kinetic\;model:\frac{M_{t}}{M_{\infty }}={\;k}_{0}t+C_{0}$$

(5) $$First-order\;kinetic\;model:Ln\left(1-\frac{M_{t}}{M_{\infty }}\right)=-k_{1}t+C_{1}$$

(6) $$Korsmeyer–Peppas\;kinetic\;model:\frac{M_{t}}{M_{\infty }}=k_{HP}t^{n}$$

### 2.12. Statistical Analysis

The data analysis was performed using Excel 2010, and the findings are presented as mean ± standard deviation derived from three repeated tests. For plotting purposes, Origin 2021 software was utilized. Differences among samples were assessed through one-way analysis of variance (ANOVA) followed by Duncan’s multiple range test, conducted with SPSS 27.0 at a significance level of α = 0.05.

## 3. Results and Discussion

### 3.1. Rheological Properties of HA/Fmoc-FRGDF Binary Hydrogels

**Figure 2:**
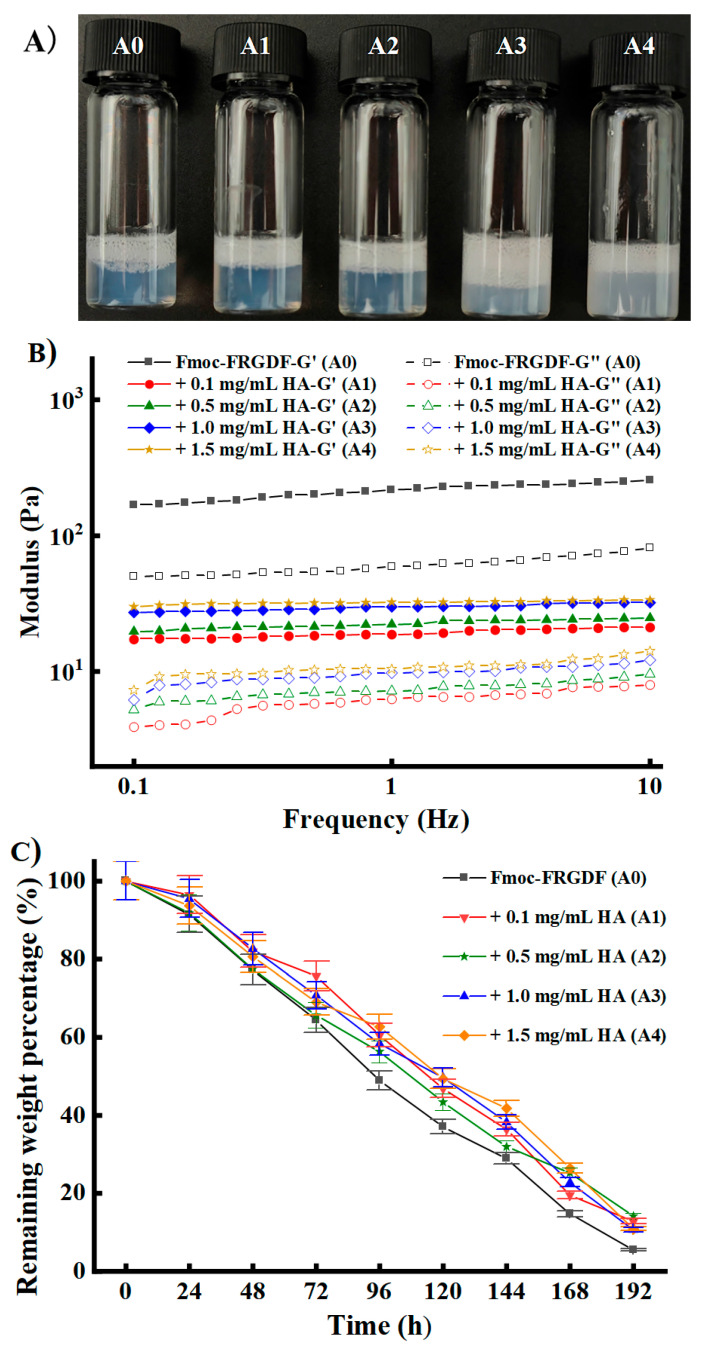
The morphology, rheological properties, and in vitro degradation of composite hydrogels. (**A**) Macroscopic pictures of the composite hydrogels (A0, A1, A2, A3, and A4 from left right). (**B**) Storage modulus (G′) and loss modulus (G″) at a shear stress of 1.0%, and (**C**) in vitro degradation curve of hyaluronic acid (HA)/Fmoc-FRGDF hydrogels (A0, A1, A2, A3, and A4).

Figure 2A shows the macroscopic morphology and physical characteristics of the hydrogels. Figure 2B illustrates the variations regarding the storage modulus (G′) and loss modulus (G”) of the HA/Fmoc-FRGDF composite hydrogels at various concentrations, subjected to a constant shear stress of 1.0% at 25.0 °C. The HA/Fmoc-FRGDF composite hydrogels exhibited viscoelastic properties, with G’ values consistently exceeding the G” values across the tested frequency range, which indicates that all HA/Fmoc-FRGDF composite systems successfully formed hydrogel structures [43]. Spanning a frequency interval from 0.1 Hz to 10.0 Hz, the Fmoc-FRGDF hydrogel exhibited the highest storage modulus (G’) at approximately 256.5 Pa. However, with an increase in the HA concentration (0.1~0.5 mg/mL), the G′ values gradually increased, although they were all lower than that of Fmoc-FRGDF, which meant that the co-assembly of HA with the SAP reduced the rheological properties of the composite nanohydrogels within a defined concentration interval of 0.1~1.5 mg/mL. This result was different with other polysaccharides, such as fucoidan [44], chitosan [19], and agarose [45], which all increased the rheological properties of the SAP hydrogel. This could have been because of the exceptional ability of HA to retain water, which reduced the rheological characteristics in the composite hydrogel [46]. Previous studies have shown that specific rheological properties are affected by various factors, including the molecular weight and concentration of HA, the pH levels, the duration of the crosslinking process, and the ratio of crosslinking agent to polysaccharide [47]. Nevertheless, the storage modulus of the hydrogels exhibited an increasing trend with an increase in HA. This could have been due to improved interactions between HA and the peptide nanofibers during the co-assembly process [48].

### 3.2. In Vitro Degradation Properties HA/Fmoc-FRGDF Binary Hydrogels

When exploring the potential of Fmoc-FRGDF self-assembling peptide (SAP) hydrogels as delivery carriers for active ingredients, their stability is of crucial importance. The effect of HA on this stability was evaluated through in vitro degradation experiments (Figure 2C). Interestingly, although the co-assembly of HA and Fmoc-FRGDF slightly reduced the initial rheological properties of the composite hydrogel, it significantly slowed down the degradation rate of the hydrogel in PBS, similar to enhancing the structural maintenance ability of food colloids in storage or digestion environments. Specifically, the degradation rate of the single Fmoc-FRGDF hydrogel was the fastest during the entire degradation process. At 96 h, the residual weight of A0 was 48.9% (*w/w*), while that of A1, A2, A3, and A4 was 60.5%, 56.3%, 58.4%, and 62.7% (*w/w*), respectively. At the end, the residual weight of A0 was only 5.5% (*w/w*) at 192 h, while the residual weights of A1, A2, A3, and A4 were 12.8%, 14.1%, 10.6%, and 10.9% at 192 h, respectively. The residual amounts of all HA/Fmoc-FRGDF composite hydrogels were found to be higher than those observed in A0. Therefore, the addition of HA effectively enhanced the stability of the Fmoc–SAP hydrogel network and prolonged the duration of its structure in the simulated physiological or digestive environment. The observed phenomenon may be attributed to the fact that each carboxylic group in the HA structure maintains a negative charge under physiological conditions. These anionic carboxyl groups, in conjunction with the acetamido functional groups, promote extensive hydrogen bonding networks with water molecules. This molecular interaction consequently stabilizes the secondary structure of the composite hydrogel [49]. This enhanced stability is particularly crucial for food applications, as it can better control the release rate of active compounds encapsulated in the gel network, achieving more sustained and controllable delivery, thereby enhancing the efficacy of such hydrogels as a carrier platform for active substances or an oral supplement.

### 3.3. The Effect of Inclusion of Que on the Properties of the Ternary Complex Hydrogels

#### 3.3.1. Rheological Properties

**Figure 3:**
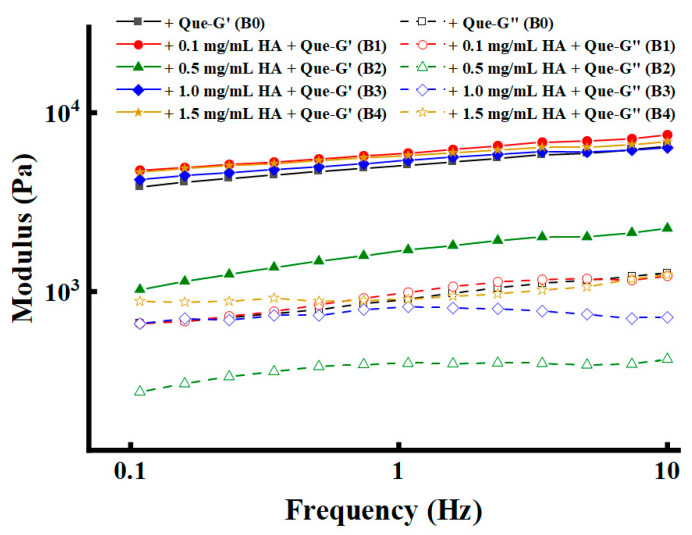
Rheological properties of composite hydrogels. Storage modulus (G′) and loss modulus (G″) at a shear stress of 1.0% of HA/Fmoc-FRGDF hydrogels (B0, B1, B2, B3, and B4) loaded with 2.0 mg/mL quercetin (Que).

The introduction of Que as a functional food ingredient or natural additive played a key role in the co-assembly of ternary (Que/HA/Fmoc-FRGDF) composite hydrogels, significantly enhancing their mechanical strength (Figure 3). This enhancement of mechanical properties directly optimizes their functional potential as carriers for the efficient and continuous release of food active ingredients [50]. Firstly, the addition of Que into the single Fmoc-FRGDF greatly increased the G′ value of the two-component hydrogel from 256.5 Pa to 6485.0 Pa. Meanwhile, the addition of Que to the HA/Fmoc-FRGDF hydrogels further increased the G′ value of the ternary complex, and sample B1 had the highest G′ value (~7527.0 Pa). The incorporation of Que notably enhanced the G′ value of the ternary complex in comparison to that of the pure Fmoc-FRGDF hydrogel and HA/Fmoc-FRGDF two-component hydrogels (Figure 2B). Furthermore, the G′ value of the ternary complexes was consistently greater than that of the Que/Fmoc-FRGDF two-component hydrogel, indicating that the interactions among the three components, Que, HA, and Fmoc-FRGDF, all contributed to the rheological properties. This interaction mechanism is consistent with the phenomenon observed in food science: phenolic compounds are prone to bind to proteins, and the interaction between polyphenols and proteins has been proven to significantly alter the rheological properties of protein–polysaccharide complex systems [51,52,53]. Therefore, the co-assembly of Que not only endows the hydrogel with a stronger mechanical stability, but also simulates the principle of polyphenols enhancing the texture in natural food systems, providing strong support for the design of advanced delivery systems with controllable release characteristics.

#### 3.3.2. Secondary Structure

**Figure 4:**
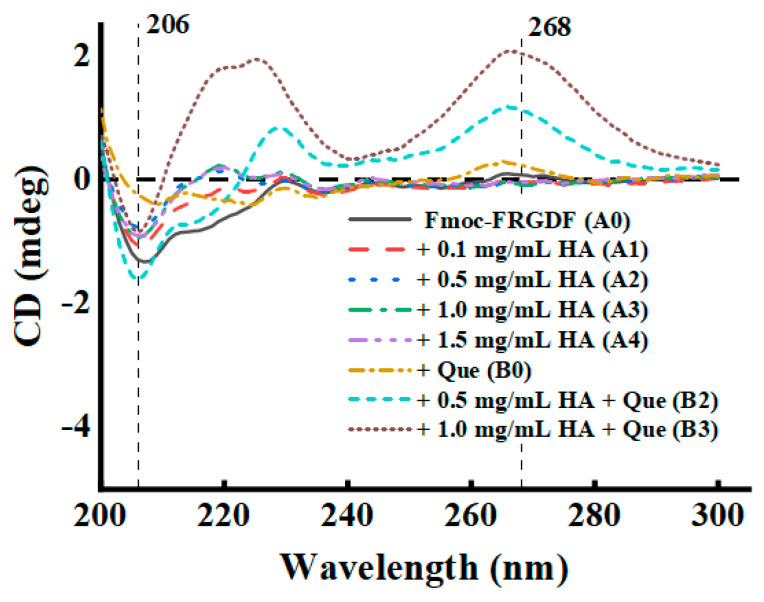
Circular dichroism (CD) spectra of HA/Fmoc-FRGDF hydrogels (A0, A1, A2, A3, and A4), and HA/Fmoc-FRGDF hydrogels (B2 and B3) loaded with 2.0 mg/mL Que.

**Figure 5:**
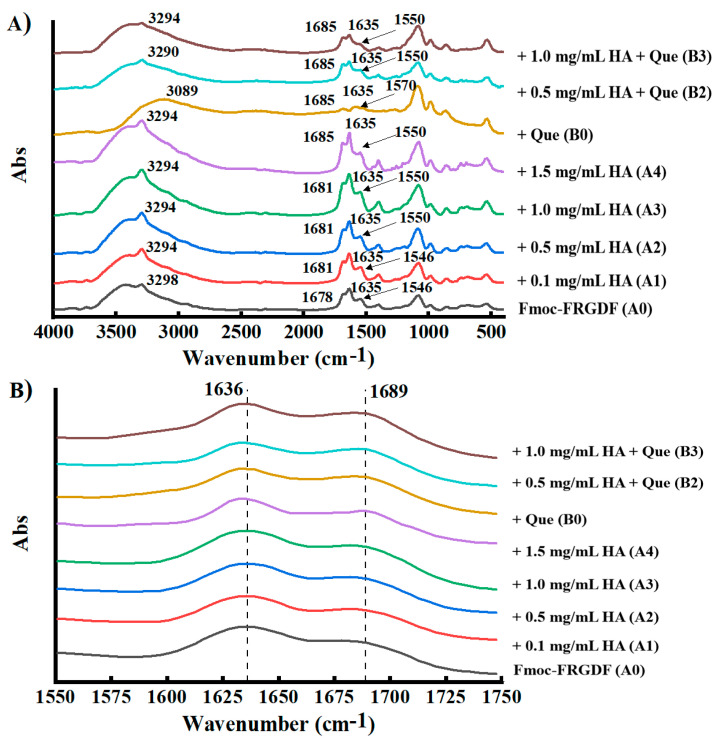
Secondary structure of composite hydrogels. (**A**) Fourier transform infrared spectroscopy spectra and (**B**) local map within the frequency range from 1550 to 1750 cm^−1^.

As a natural polyphenol functional factor, the co-assembly of Que had a key regulatory effect on the secondary structure and supramolecular order of the Que/HA/Fmoc-FRGDF ternary composite hydrogel, significantly improving its performance as a delivery carrier (Figure 4 and Figure 5). Figure 4 presents the circular dichroism spectra in the range of 200~300 nm for a more detailed examination of the hydrogel’s secondary structure. The peak of the Que/HA/Fmoc-FRGDF hydrogel (B2 and B3) was widened and shifted to a lower wavelength in the range of 200~220 nm, indicating that the co-assembly of Que and HA/Fmoc-FRGDF may strengthen the β-sheet structure [44]. Previous study has also demonstrated that when a self-assembled gel adopts a β-sheet configuration, intermolecular interactions enhance its stability, which is reflected in an increase in the rheological G′ value [54]. Furthermore, wide transitions of samples B2 and B3 were observed between 230 and 270 nm, attributed to π-π transitions, suggesting the presence of supramolecular interactions among Que, HA, and the nanofibers [55]. Supramolecular ordering refers to the process in which macromolecules spontaneously form ordered structures through non-covalent bond interactions [44]. In this case, they could be considered as three distinct macromolecules—a long polymer of Fmoc-FRGDF, HA and Que—but together they formed a single entity. It is the intermolecular attraction between these three polymers that drives the formation of this single entity. In particular, compared with the single Fmoc-FRGDF hydrogel, the peak intensity at 230~270 nm of the Que/HA/Fmoc-FRGDF (B2 and B3) hydrogel was significantly enhanced, indicating that the addition of Que promoted supramolecular ordering. Previous studies have established this effect as a stable fabrication technique between two polymers [16,44,56]. This reveals that Que, HA, and Fmoc-FRGDF formed a highly ordered supramolecular network through non-covalent interactions (such as π-π packing and hydrophobic interactions).

The FTIR spectra showed that all samples exhibited N-H and O-H stretching vibration peaks of the amide A band at 3294 cm^−1^, and the addition of HA enhanced the peak intensity (Figure 5A). This may have been due to the abundant OH groups in HA molecules [57]. Furthermore, the co-assembly of HA with Fmoc-FRGDF led to a distinct red shift of the O-H peak from 3425 cm^−1^ (Fmoc-FRGDF) to 3375 cm^−1^ (A3), indicating the presence of hydrogen bonds between the amino group of Fmoc-FRGDF and the hydroxyl group of HA [48]. Similarly, the O-H peak of the Que/HA/Fmoc-FRGDF ternary complex shifted to 3089 cm^−1^ (B0) and 3383 cm^−1^ (B2) from 3425 cm^−1^ for Fmoc-FRGDF, which also suggested hydrogen bonding among Que, HA, and Fmoc-FRGDF [48]; the peaks became less prominent, indicating that the binding of Que to Fmoc-FRGDF and/or HA restricted molecular vibration and stretching [58]. The amide I peaks shifted to 1635~1685 cm^−1^ for A2–A4, compared to 1635~1678 cm^−1^ for A0. Additionally, these peaks shifted to 1660 cm^−1^ for Que from 1685 cm^−1^ for B0; the amide II peaks also exhibited a shift to 1550 cm^−1^ for A2–A4 from an original position of 1546 cm^−1^ for A0, and similarly shifted to 1550 cm^−1^ for B2–B3 from 1597 cm^−1^ for B0. These shifts indicate the presence of hydrophobic interactions and hydrogen bonding among Que, HA, and SAP [59]. Previous research has also shown that polyphenols can bind to proteins through hydrophobic interaction between the aromatic ring of polyphenols and the hydrophobic amino acids of proteins [60].

Figure 5B emphasizes the amide I region from 1600 to 1700 cm^−1^, which is frequently utilized to characterize the properties of secondary structures created by peptide sequences. The FTIR spectra of the pure SAP hydrogel, HA/Fmoc-FRGDF two-component hydrogel, and Que/HA/Fmoc-FRGDF ternary component hydrogel were similar, which exhibited peaks at 1689 cm^−1^ and 1636 cm^−1^, respectively, indicating the presence of a β-sheet structure [59]. Meanwhile, the co-assembly of Que and HA did not disrupt the β-sheet structure of the hydrogels. Previous research has demonstrated that the positively charged amino acid arginine exhibits the strongest binding affinity towards negatively charged HA, highlighting the importance of electrostatic interactions and side-chain specificity within these complexes. Furthermore, HA’s carboxyl and amide groups contribute significantly to its interaction with peptides, which is further stabilized by hydrogen bonds [61]. This phenomenon may also have been present in the Que/HA/Fmoc-FRGDF ternary complexes. This dense supramolecular network, constructed and driven by Que, based on the β layer skeleton and reinforced by multiple non-covalent bonds (hydrogen bonds, hydrophobic interactions, and π-π interactions), not only greatly enhanced the mechanical strength of the hydrogel, but more importantly, provided a highly stable microenvironment for the food active substances encapsulated within it. Ensuring that it can achieve more precise, longer lasting, and more controllable release during processing, storage, and even digestion lays a solid material foundation for the development of the next generation of high-performance intelligent delivery systems.

### 3.4. Microscopic Structure Observation

**Figure 6:**
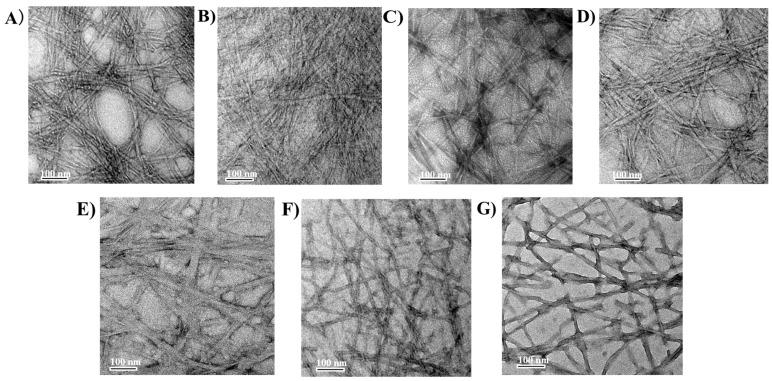
Images of hydrogels captured using TEM. (**A**) Fmoc-FRGDF (A0); (**B**) +0.1 mg/mL HA (A1); (**C**) +0.5 mg/mL HA (A2); (**D**) +1.0 mg/mL HA (A3); (**E**) +1.5 mg/mL HA (A4); (**F**) +0.5 mg/mL HA+Que (B2); and (**G**) +1.0 mg/mL HA + Que (B3).

TEM images visually reveal the fine nanofiber networks and their bundle structures formed inside the HA/Fmoc-FRGDF and Que/HA/Fmoc-FRGDF composite hydrogels (Figure 6). This microscopic morphology is crucial for their performance as delivery carriers. Figure 6A–E show that HA/Fmoc-FRGDF formed a dense network structure. When the HA content was 1.5 mg/mL (A4), obvious fiber bundles formed, and these thicker fiber bundles might have resulted in the higher G′ of the hydrogel. This means that the hydrogel acquires a greater mechanical strength, which enables it to better resist physical stress during food processing and protect the sensitive active ingredients (such as vitamins) encapsulated within from damage [62]. The diameter of the nanofibers was measured using ImageJ, and the average diameters of each sample were A0, 12.8 ± 1.4 nm; A1, 13.7 ± 0.4 nm; A2, 15.7 ± 0.4 nm; A3, 16.5 ± 0.4 nm; A4, 31.2 ± 1.7 nm; B2, 16.1 ± 0.5 nm; and B3, 20.1 ± 2.71 nm. It is worth noting that the introduction of Que did not destroy the intrinsic structure of the nanofibers (Figure 6F,G). The observed nanofibers resulted from hierarchical self-assembly driven by the π-π stacking of Fmoc aromatic rings, hydrogen bonding between HA carboxyl groups and FRGDF amide bonds, electrostatic forces, and hydrophobic interactions [16,41,63]. Fmoc-FRGDF has been co-assembled with chitosan [64] and agarose [45] to form hydrogels. TEM characterization revealed that these binary systems generated nanofibers through electrostatic-driven assembly. This microstructure, on the one hand, indicates that the composite hydrogel ensures the uniform distribution and structural integrity of active molecules such as Que within the carrier; on the other hand, its dense fiber network can effectively delay the diffusion rate of the encapsulated functional factors to the food substrate [65], thereby achieving more persistent and controllable release characteristics. Therefore, the TEM images confirm the superior microstructure of the composite hydrogel, providing a solid morphological basis for its application potential as a high-performance delivery carrier of nutritional active substances.

### 3.5. Encapsulation and In Vitro Release of Que

**Figure 7:**
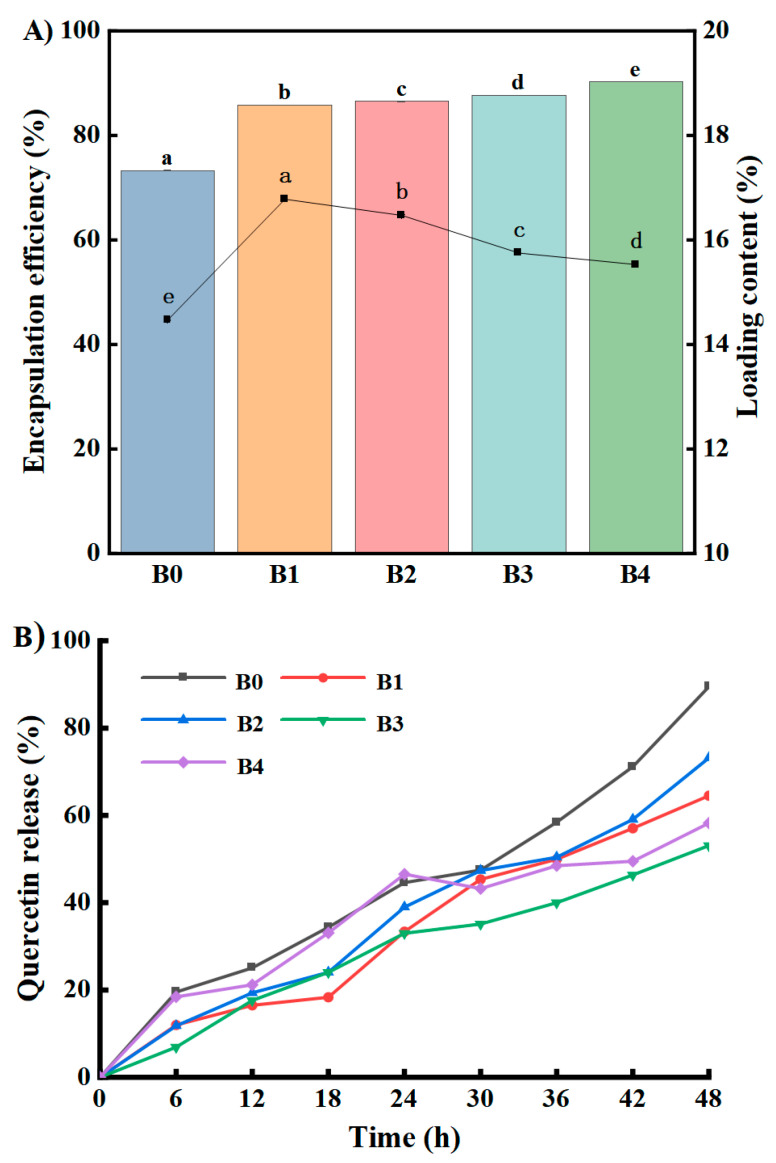
The encapsulation efficiency (EE%) and loading capacity (LC) (**A**), (**B**) in vitro controlled release behavior of quercetin (Que) encapsulated in HA/Fmoc-FRGDF hydrogels at HA concentrations of 0, 0.1, 0.5, 1.0, and 1.5 mg/mL. B0–B4: Que/HA (0, 0.1, 0.5, 1.0, and 1.5 mg/mL)/Fmoc-FRGDF (10 mg/mL) hydrogel. Different letters showed significant differences at α=0.05.

The self-assembling peptide Fmoc-FRGDF, verified by cytotoxicity, is non-toxic to normal oral epithelial cells [16]. Bellar et al. [66] demonstrated that the oral administration of HA35 (140 mg/day) is a safe treatment for healthy individuals, without affecting metabolism, inflammation, or microbiome parameters, demonstrating its good biocompatibility. When further used as a delivery carrier of Que, this system demonstrated a high stability and provided safety guarantees for subsequent in vivo applications. The excellent encapsulation efficiency (EE%) and controllable release characteristics exhibited by Que in the HA/Fmoc-FRGDF composite hydrogel provide key performance support for its use as a delivery carrier of nutritional active substances. Figure 7A demonstrates the encapsulation efficiency (EE%) of Que that was loaded into the HA/Fmoc-FRGDF hydrogels. The results show that the EE% of Que loaded into the four HA/Fmoc-FRGDF hydrogels ranged from 85.8% to 90.3%, significantly higher than that of the Fmoc-FRGDF hydrogel (73.3%) (*p* < 0.05). This means that during food processing or carrier preparation, more Que, with health benefits but prone to loss, can be effectively captured and stably present in the gel network, reducing activity loss and increasing the functional component content of the final product. Figure 7B shows that Que had an LC ranging from 15.5% to 16.8% of all hydrogels. The release rate of Que in the single Fmoc-FRGDF hydrogel (B0) was 87.4% at 48 h, while the release rate of Que loaded in samples B1–B4 was lower than that of B0 at 48 h, at 64.9%, 73.5%, 53.8%, and 58.0%, respectively (Figure 7A). This result shows that the co-assembly of HA with Fmoc-FRGDF had a controlled Que release effect, although the co-assembly of HA decreased the hydrogel’s rheological properties (Figure 2B). This might have been due to the addition of HA improving the strength of non-covalent interactions between the HA and Fmoc-FRGDF molecules and the stability of the composite hydrogels. Interestingly, a reabsorption phenomenon and decreased release rate were observed in sample B4 between 24 and 30 h. This may be attributed to the dense network structure (nano-bundles) of the sample (Figure 6E). Additionally, the intramolecular interaction forces may change, leading to altered release and absorption behaviors with the degradation of the hydrogel [67,68]. This may demonstrate the potential of the intelligent responsiveness of composite hydrogels in food delivery (adapting to complex digestive environments).

In order to precisely design and optimize the release behavior of the active components of the Que/HA/Fmoc-FRGDF hydrogel, it is crucial to understand its release kinetics. The release or dissolution of drugs is a multifaceted process that can be described by various mathematical models. The commonly used in vitro release kinetics models include zero-order, first-order, and third-order kinetics, as well as those proposed by and Korsmeyer–Peppas [42]. The zero-order drug delivery system represents a controlled approach to drug administration, which has the potential to enhance and optimize the efficacy of medications [69,70]. In contrast, first-order kinetics indicate that the concentration of a drug (typically represented on the *y*-axis) increases linearly (i.e., in direct proportion) with dosage (usually depicted on the *x*-axis). This relationship suggests that within first-order pharmacokinetics, target occupancy is directly correlated with an increase in drug dosage [71]. To further investigate the release characteristics of the hydrogels, these three distinct kinetic models were employed to determine the kinetic parameters (Table 1), and the mathematical model with an R^2^ value closest to one was chosen as the drug release kinetic model.

As shown in Table 1, the Prob > F for all samples across different dynamic models was found to be less than 0.05, indicating significant differences in all fitted curves at a 95.0% confidence level (*p* < 0.05). Furthermore, the R^2^ values for both the Korsmeyer and Peppas kinetic models were the highest among all samples, suggesting that the Que release kinetics aligned well with the models proposed by Korsmeyer and Peppas. Notably, Que release at HA concentrations of 0.5 mg/mL obeyed both zero-order and Korsmeyer and Peppas’ kinetic models. In addition, the value of “*n*” in the formula $\frac{{\;M}_{t}}{M_{\infty }}\;=\;k_{HP}t^{n}$ represents a distinct release mechanism [72]. According to Table 1, the diffusion index “n” for the samples ranged from 0.5 to 1, suggesting that Que release occurred through a non-Fickian process or was characterized by anomalous diffusion.

## 4. Conclusions

The co-assembly of Que, HA, and Fmoc-FRGDF peptide successfully constructed a high-performance delivery platform. Its core advantage lies in significantly improving the performance of the carrier while retaining the inherent π-β supramolecular assembly mechanism driven by hydrogen bonds, electrostatic interactions, and hydrophobic interactions. The addition of HA significantly improved the encapsulation efficiency of functional polyphenol Que and ensured its stability during processing and storage. Notably, co-assembly with Que reinforced β-sheet structures and significantly promoted supramolecular ordering. On the other hand, by the means of physical barriers and strengthening intermolecular forces, the in vitro release rate of Que could be effectively delayed, which is crucial for precisely regulating the delivery sequence of drugs and/or bioactive ingredients in the digestive tract. The pharmacokinetic behavior of quercetin is mainly governed by the kinetics of Korsmeyer and Peppas; its release occurs through non-Fick mechanisms or abnormal diffusion processes. The precise regulation of physical parameters by using natural ionic polysaccharides such as HA provides a highly promising strategy for delivering hydrophobic active ingredients in nutritional supplements and/or medicine. However, the in vivo applications of this hydrogel system have not been explored. Although the current Fmoc-FRGDF/HA system is not food-compliant, it establishes a versatile platform for developing food-compatible hydrogel systems for subsequent food applications. This efficient assembly methodology empowers the engineering of sustainable food-grade delivery systems with tunable functionalities, such as targeted delivery, the controlled release of flavor substances, and nutrient preservation. Future work will explore food-grade RGD peptides from soy or seaweed sources (e.g., phycobiliproteins) to create GRAS-qualified delivery systems for functional foods like antioxidant-fortified beverages or pH-protective nutrition gels.

## Figures and Tables

**Table 1:** Parameters related to the four kinetic models concerning the release properties of hesperidin from all hydrogel systems.

Sample		Zero-Order	First-Order	Korsmeyer and Peppas
**B0**	Fitted curve	$\frac{Mt}{M\infty }=0.6096t-2.2888$	$\frac{Mt}{M\infty }=-210.384(1-e^{0.0025t})$	$\frac{Mt}{M\infty }=2.8669t^{0.8611}$
	R^2^	0.9727	0.9717	0.9737
	Prob > F	4.1207 × 10^−7^	3.2795 × 10^−8^	1.5263 × 10^−8^
**B1**	Fitted curve	$\frac{Mt}{M\infty }=0.7254t-1.1481$	$\frac{Mt}{M\infty }=-31091(1-e^{-0.00004t})$	$\frac{Mt}{M\infty }=2.0747t^{0.8946}$
	R^2^	0.9879	0.9850	0.9906
	Prob > F	3.6121 × 10^−8^	2.7540 × 10^−9^	5.3644 × 10^−10^
**B2**	Fitted curve	$\frac{Mt}{M\infty }=0.6808t-0.4521$	$\frac{Mt}{M\infty }=-767(1-e^{-0.0019t})$	$\frac{Mt}{M\infty }=1.6900t^{0.9639}$
	R^2^	0.9901	0.9800	0.9901
	Prob > F	1.7698 × 10^−8^	1.3585 × 10^−8^	7.5763 × 10^−10^
**B3**	Fitted curve	$\frac{Mt}{M\infty }=0.8910t-2.0717$	$\frac{Mt}{M\infty }=-56.6366(1-e^{0.0114t})$	$\frac{Mt}{M\infty }=2.27685t^{0.8181}$
	R^2^	0.9829	0.9928	0.9930
	Prob > F	1.2025 × 10^−7^	2.7232 × 10^−10^	1.5440 × 10^−10^
**B4**	Fitted curve	$\frac{Mt}{M\infty }=0.8523t-5.8488$	$\frac{Mt}{M\infty }=-11.3706(1-e^{0.0293t})$	$\frac{Mt}{M\infty }=7.1637t^{0.5294}$
	R^2^	0.8720	0.9276	0.9554

Note: B0–B4: Que/HA (0, 0.1, 0.5, 1.0, and 1.5 mg/mL)/Fmoc-FRGDF (10 mg/mL) hydrogel.

## Data Availability

The original contributions presented in the study are included in the article, further inquiries can be directed to the corresponding author.
